# A Novel Long-Term Tympanostomy Tube: The U-Tube

**DOI:** 10.3390/bioengineering13010079

**Published:** 2026-01-12

**Authors:** Itay Chen, Jean-Yves Sichel, Chanan Shaul, Ronen Perez

**Affiliations:** Department of Otolaryngology-Head and Neck Surgery, Shaare Zedek Medical Center, Faculty of Medicine, Hebrew University of Jerusalem, Jerusalem 99308, Israel

**Keywords:** tympanostomy tube, ventilation tube, otitis media, perforation rate, insertion time, middle ear ventilation

## Abstract

Purpose: Tympanostomy tubes are essential for middle ear ventilation, but conventional long-term tubes carry high perforation rates (12–22%). This study evaluated the Tympanostomy U-Tube (TUT), a novel silicone-based tube designed to minimize perforation risk by redistributing pressure away from the tympanic membrane rim. Methods: This was a retrospective cohort study of 192 ears in children aged 1–4 years who underwent TUT insertion for chronic otitis media with effusion or recurrent acute otitis media. The primary outcomes were tube insertion time and the permanent perforation rate. Mean follow-up was 38.4 months. Results: Mean tube insertion time was 21.6 months. Spontaneous extrusion occurred in 18.2% of ears (mean 24.5 months), while 81.8% underwent elective removal (mean 21.0 months). Permanent perforation developed in only 4 ears (2.08%; 95% CI: 0.6–5.2%), substantially lower than rates reported in the literature for conventional long-term tubes (12–22%), although the retrospective design and reliance on historical controls limit direct comparison. Complications were minimal, with otorrhea (36%) responding to topical therapy. Office-based removal was successful in all cases. Conclusions: The TUT provides intermediate-duration ventilation with a perforation rate comparable to that of short-term tubes, while avoiding the high perforation rates of conventional long-term tubes. Prospective randomized trials are needed to validate these findings.

## 1. Introduction

Otitis media (OM) represents one of the most common pediatric infectious diseases worldwide, with approximately 80% of children experiencing at least one episode by school age [1,2]. Among its various manifestations, otitis media with effusion (OME) is particularly prevalent, affecting between 80–90% of children before they reach school age [2,3]. The peak incidence occurs between 6 and 24 months of age, with prevalence rates ranging from 4.3% to 14.2% in school-aged children [4,5,6]. The unpredictable course of OME makes it a major pediatric health concern [7,8,9].

Tympanostomy tube (TT) insertion has become the most common ambulatory surgical procedure performed on children in the United States, with approximately 9% of children requiring tube placement [10]. These tubes serve to ventilate the middle ear, equalize pressure, prevent fluid accumulation, and provide a route for topical antibiotic delivery [11,12]. Clinical practice guidelines have established clear indications for TT insertion, including chronic OME persisting for three months or longer with documented hearing difficulties [7,10]. International consensus guidelines have further emphasized individualized decision-making based on hearing status and developmental considerations [8].

Despite widespread use, conventional TTs have limitations, including suboptimal ventilation duration and a risk of persistent tympanic membrane perforation [13,14,15,16]. Short-term tubes typically remain in place for 8–10 months while long-term tubes last 32.8–34.8 months [17,18]. However, permanent perforation rates range from 12% to 22% for long-term tubes, compared with 2% to 6% for short-term tubes [13,15,19].

The materials used in TT manufacturing have evolved from metal to predominantly silicone or fluoroplastic [20]. Recent studies demonstrate that silicone tubes have lower rates of post-operative otorrhea than fluoroplastic alternatives [21]. A comprehensive review of tympanostomy tube designs highlights the importance of flange configuration and material composition in determining clinical outcomes [22]. A critical aspect of TT design is the interaction at the perforation site. Conventional tubes apply direct pressure on the rim, potentially compromising local blood supply [23].

**Figure 1:**
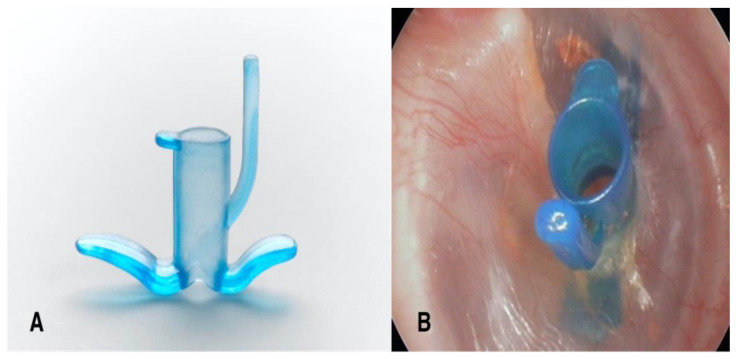
The Tympanostomy U-Tube (TUT). (**A**) The TUT features a unique U-shaped design with arched, flexible arms, a conical shaft, and an extraction handle (tail). (**B**) TUT inserted in the right tympanic membrane, demonstrating proper positioning with both flanges visible.

In the past decade, the Tympanostomy U-Tube (TUT) was introduced (Figure 1), specifically engineered to provide adequate long-term ventilation while minimizing perforation risk. The TUT employs a unique arched silicone structure designed to reduce direct pressure on the perforation rim. Unlike conventional tubes, where flanges directly contact and compress the perforation rim, the TUT’s arched arms maintain approximately 1 mm distance from the rim, with only small flat pedicles providing contact, thereby theoretically redistributing pressure away from critical vascular structures and potentially preserving local blood supply. This study evaluates the safety and device performance of the TUT, with particular focus on insertion time and the rate of permanent perforation. Functional hearing outcomes and patient-reported measures were beyond the scope of this analysis.

## 2. Materials and Methods

### 2.1. Study Design

**Figure 2:**
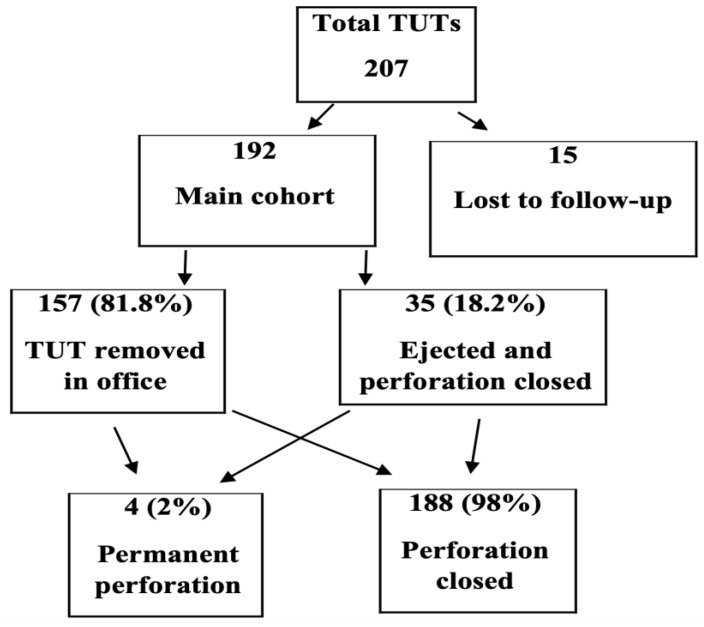
Study flow chart. Of 207 ears treated with Tympanostomy U-Tube (TUT), 15 were lost to follow-up. Among the 192 ears in the main cohort, 157 (81.8%) underwent elective removal in the office, while 35 (18.2%) were spontaneously ejected with perforation closure. Only 4 ears (2%) developed permanent perforation.

This retrospective cohort study was conducted at Shaare Zedek Medical Center, a tertiary referral center for pediatric otolaryngology in Jerusalem, Israel. The study protocol was approved by the Institutional Helsinki Committee (Ethics Committee). All clinical charts of children who underwent TUT insertion between 2012 and 2020 were reviewed. Inclusion criteria were age 1–4 years, diagnosis of chronic OME (≥3 months) or recurrent acute otitis media, and a minimum 12-month follow-up. Only isolated TUT insertion procedures were included; cases involving concurrent adenoidectomy, adenotonsillectomy, or other surgical interventions were excluded to minimize potential confounding factors Exclusion criteria included craniofacial anomalies that may congenitally affect Eustachian tube function (e.g., cleft lip and/or palate, Pierre Robin sequence), Down syndrome (trisomy 21), prior tympanostomy tubes, and baseline tympanic membrane perforation. Of 207 ears treated, 15 (7.2%) were lost to follow-up, leaving 192 ears for analysis (Figure 2).

### 2.2. Surgical Procedure

All TUT insertions were performed under general anesthesia by experienced pediatric otolaryngologists. Following microscopic examination, a myringotomy incision was made in the anteroinferior quadrant, middle ear fluid was aspirated when present, and the TUT was inserted using standard forceps. Proper positioning was confirmed by visualization of both flanges. Post-operatively, patients were followed at 3-month intervals for the first year, then every 6 months until tube extrusion or removal.

### 2.3. Description of Tympanostomy U-Tube

**Figure 3:**
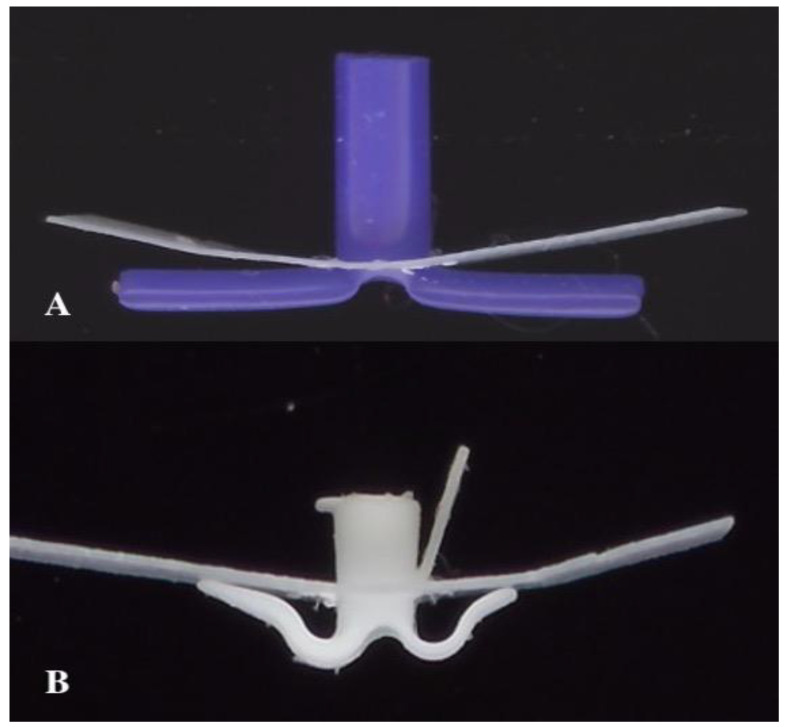
Biomechanical design comparison. (**A**) Conventional long-term tympanostomy tube (T-tube) showing direct flange contact with the perforation rim. (**B**) Tympanostomy U-Tube (TUT) demonstrating the arched arms maintaining distance from the perforation rim, with only small pedicles in contact, redistributing pressure away from the critical healing zone.

The TUT (Otomedics, Jerusalem, Israel) is manufactured from flexible, springy medical-grade silicone. The distinctive design features arched arms positioned so that their contact pedicles maintain approximately 1 mm from the perforation rim, redistributing mechanical forces away from the immediate perforation edge and, theoretically, preserving local blood supply (Figure 3). The internal diameter is 1.3 mm with a conical shaft facilitating drainage. An extraction handle and posterior arm slit enable painless office-based removal: gentle traction causes the posterior arm to fold inward, allowing atraumatic passage through the perforation.

### 2.4. Outcome Measures

Primary outcomes were (1) tube insertion time (interval between placement and extrusion/removal) and (2) permanent perforation rate (perforation persisting > 6 months post-tube). Secondary outcomes included spontaneous extrusion versus elective removal rates, post-operative complications, and need for tube replacement. TUT outcomes were compared descriptively with published data on conventional tympanostomy tubes from systematic reviews and meta-analyses.

### 2.5. Statistical Analysis

Continuous variables are presented as means ± standard deviation. Categorical variables are reported as frequencies and percentages. Tube insertion times for spontaneously extruded and electively removed TUTs were compared using an independent-samples *t*-test. Perforation rates were calculated with 95% confidence intervals. The association between tube removal method and perforation risk was assessed using Fisher’s exact test. Given the low number of outcome events (*n* = 4 perforations), risk factor analyses were exploratory and limited to univariate comparisons. Multivariable logistic regression was not performed due to insufficient events to support stable parameter estimation. Statistical significance was defined as *p* < 0.05. Analyses were performed using SPSS Statistics version 25.0 (IBM Corp., Armonk, NY, USA).

## 3. Results

### 3.1. Study Population and Demographics

Following application of inclusion and exclusion criteria, 192 ears from 114 patients were included in the final analysis. The patient cohort comprised 67 males (58.8%) and 47 females (41.2%), with a mean age at surgery of 2.8 ± 0.9 years (range: 1.0–4.0 years). Bilateral tube insertion was performed in 78 patients (68.4%), while 36 patients (31.6%) underwent unilateral insertion. The primary indication for surgery was chronic otitis media with effusion in 156 ears (81.3%) and recurrent acute otitis media with persistent effusion in 36 ears (18.7%). The mean follow-up duration was 38.4 ± 14.2 months.

### 3.2. Tube Insertion Time and Extrusion Patterns

The overall mean insertion time for TUT was 649 ± 186 days (21.6 months; median: 638 days). Of the 192 ears analyzed, spontaneous extrusion occurred in 35 ears (18.2%), while elective removal was performed in 157 ears (81.8%). The insertion time differed significantly between these two groups: spontaneously extruded tubes remained in situ for a mean of 735 ± 142 days (24.5 months), whereas electively removed tubes had a mean insertion time of 630 ± 188 days (21.0 months) (*p* = 0.002). Analysis by age group revealed that younger children demonstrated longer tube retention times. The distribution of tube insertion times is presented in Table 1.

Elective removal was performed based on clinical judgment, considering factors such as age progression beyond the high-risk period for OME (typically 4–5 years), tube-related complications such as persistent otorrhea attributed to tube presence, and parental preference following an extended asymptomatic period. The surgeon’s assessment ultimately guided the decision that the probability of effusion recurrence was sufficiently low to warrant tube removal. All elective extractions were performed in the office setting without anesthesia, using the TUT extraction handle for painless, atraumatic removal.

### 3.3. Tympanic Membrane Perforation Rates

Among the 35 ears with spontaneous tube extrusion, complete perforation closure occurred in 34 ears (97.1%), with only one ear (2.9%) demonstrating persistent perforation. In the elective removal group (157 ears), the perforation closed spontaneously in 154 ears (98.1%), while three ears (1.9%) developed persistent perforations. The overall permanent perforation rate across all 192 ears was 2.08% (4/192; 95% CI: 0.6–5.2%). Notably, two of the four persistent perforations occurred in the same patient. The mean insertion time for ears that developed persistent perforation was 31.5 ± 2.4 months versus 21.2 ± 5.8 months for those that healed (*p* = 0.004). In exploratory univariate analysis, tubes remaining in place beyond 30 months demonstrated higher perforation rates (5.8% vs. 0.7%, *p* = 0.035). However, due to the small number of perforation events (*n* = 4), multivariable analysis adjusting for potential confounders, including age, indication, laterality, and surgeon factors, was not feasible.

### 3.4. Comparison with Conventional Tympanostomy Tubes

Table 2 presents a comparison of TUT outcomes with published data on conventional short-term and long-term tympanostomy tubes. The TUT demonstrated intermediate insertion time characteristics, remaining in place longer than short-term tubes but for a shorter duration than traditional long-term tubes. Notably, the permanent perforation rate of the TUT (2.08%) was comparable to that of short-term tubes (2–6%) and substantially lower than rates reported in the literature for long-term tubes (12–22%). However, these comparisons are indirect and subject to heterogeneity in study designs, patient populations, and follow-up protocols.

## 4. Discussion

This retrospective study evaluated the clinical outcomes of the Tympanostomy U-Tube (TUT), a novel silicone-based ventilation tube designed to minimize the risk of perforation by biomechanically redistributing pressure away from the tympanic membrane perforation rim. The results demonstrate that the TUT achieved a mean insertion time of 21.6 months and a permanent perforation rate of 2.08%, positioning it as an intermediate-duration tube with a safety profile comparable to that of short-term tubes. This study was designed to evaluate the safety and device performance of the TUT, with a focus on perforation rates and insertion time. Functional hearing outcomes and patient-reported measures were beyond the scope of this analysis.

It is important to note that the current study lacks a concurrent control group, and all comparisons with conventional tympanostomy tubes are based on published literature. Differences in patient selection criteria, surgical techniques, follow-up duration, and tube removal protocols across studies may confound these comparisons. Therefore, our findings should be interpreted as hypothesis-generating rather than definitive evidence of superiority.

The primary finding is the low perforation rate observed with the TUT, which contrasts favorably with published rates for conventional long-term tubes. While long-term tubes typically demonstrate perforation rates of 12–22% [15,17,18,19], the TUT’s rate of 2.08% approaches that reported for short-term tubes (2–6%) [15,20,21]. This outcome supports the hypothesis that the TUT’s design may preserve local vascularity and promote healing after tube removal. However, this was not a randomized controlled trial, and multiple factors beyond tube design may contribute to perforation rates.

The TUT insertion time (mean 21.6 months) falls between the short- and long-term tube groups [18,23,24,25]. This duration may be clinically advantageous for children requiring ventilation beyond the capacity of short-term tubes. The 81.8% elective removal rate reflects the surgeon’s ability to control tube duration, potentially mitigating the risk of perforation. The finding that tubes remaining beyond 30 months demonstrated higher perforation rates (5.8% vs. 0.7%, *p* = 0.035) underscores the importance of surveillance and timely removal.

An important practical advantage of the TUT is the feasibility of office-based removal without sedation or anesthesia. The extraction handle enables simple, painless removal via gentle traction, providing clinicians with significant flexibility in timing the procedure according to their clinical judgment. When the treating physician determines that the risk of OME recurrence is low—whether due to age progression beyond the high-risk period, resolution of symptoms, or other clinical factors—the tube can be promptly removed without the need to schedule an operating room procedure. This ability to intervene early when clinically indicated may contribute to the low perforation rate observed in our study, as prolonged tube retention is a known risk factor for persistent perforation. Additionally, this capability reduces healthcare costs and anesthetic exposure, though this benefit must be weighed against device cost [26,27,28,29]. Recent studies comparing in-office versus operating room procedures have demonstrated comparable outcomes, further supporting the feasibility of office-based approaches in appropriately selected patients [29].

Several aspects warrant cautious interpretation. First, the comparison with literature-reported outcomes is indirect and subject to heterogeneity. A prospective randomized trial would provide more definitive evidence. Furthermore, the proposed biomechanical mechanism—that the TUT’s arched design reduces direct pressure on the perforation rim and thereby preserves local vascularity—remains hypothetical. Direct validation would require studies incorporating laser Doppler flowmetry to assess tympanic membrane perfusion, or histological analysis of the healing tissue following tube removal. Until such evidence is available, the favorable perforation rates observed should not be causally attributed to the proposed mechanism.

The clinical implications of our findings extend to patient selection and management strategies. For children aged 1–2 years presenting with chronic OME, the TUT may offer particular advantages. This age group demonstrated the longest mean insertion time (23.4 months) in our cohort, potentially providing continuous ventilation through the peak years of OME prevalence (ages 1.5–4.5 years) with a single tube insertion [7,9]. This could reduce the need for repeat procedures, which carry cumulative anesthetic risks and healthcare costs. However, the decision between tube types must be individualized based on multiple factors, including disease severity, anticipated ventilation duration, family preferences, and surgeon experience. The ability to perform office-based removal represents a significant practical advantage, as studies have demonstrated comparable pain levels and family satisfaction between office and operating room removal when appropriate techniques are employed [24,30]. Future research should incorporate cost-effectiveness analyses comparing the TUT with conventional tubes, accounting for device costs, anesthesia exposure, repeat procedure rates, and complication management. Additionally, long-term audiological outcomes and quality-of-life assessments would provide valuable data for a comprehensive evaluation of the TUT’s clinical utility in pediatric OM management [7,10].

This study has several limitations. First, the retrospective design introduces potential for selection bias and unmeasured confounding. The absence of a concurrent control group precludes direct comparison with conventional tubes under identical conditions. Second, the single-center nature limits generalizability. Third, the decision to remove the elective tube was based on clinical judgment rather than standardized criteria. Fourth, follow-up duration may have been insufficient to detect late complications in some cases. Fifth, we did not systematically collect audiometric data, quality-of-life measures, or cost-effectiveness analyses. Finally, comparisons with the published literature are subject to heterogeneity in study designs and reporting standards.

An additional methodological consideration is that our study included only isolated TUT insertions, excluding cases performed concurrently with adenoidectomy or adenotonsillectomy. While this design choice allowed us to evaluate TUT outcomes without the confounding effects of concurrent procedures, it may limit the generalizability of our findings to clinical scenarios where combined surgery is indicated. The potential interaction between adenoid removal and TUT performance—particularly with respect to ventilation duration and effusion recurrence—remains an area for future investigation.

## 5. Conclusions

In this retrospective cohort, the Tympanostomy U-Tube demonstrated a low rate of permanent perforation (2.08%) and an intermediate duration of ventilation, averaging 21.6 months. The design facilitates office-based removal and showed acceptable complication rates. Whether the favorable outcomes are attributable to the TUT’s unique biomechanical design or to other factors such as patient selection and follow-up protocols remains to be determined. Prospective randomized controlled trials are needed to validate these findings and directly compare the TUT with standard tubes under controlled conditions. Until such evidence becomes available, the TUT represents a reasonable option for children requiring intermediate-duration middle-ear ventilation, particularly when combined with regular surveillance and timely removal to minimize the risk of perforation.

## Figures and Tables

**Table 1:** Distribution of Tube Insertion Times.

Insertion Time (Months)	Number of Ears	Percentage (%)
<12	16	8.3
12–18	34	17.7
18–24	47	24.5
24–30	43	22.4
30–36	18	9.4
>36	34	17.7

**Table 2:** Comparison of Tympanostomy U-Tube (TUT) with Conventional Tympanostomy Tubes.

**Tube Type**	**Mean Insertion Time (Months)**	**Perforation Rate (%)**	**References**
Short-term tubes	8–10	2–6	[13,14,15,19,20,21]
Long-term tubes	32.8–34.8	12–22	[15,17,18,19]
TUT (current study)	21.6	2.08 (95% CI: 0.6–5.2)	Current study

## Data Availability

The data presented in this study are available on request from the corresponding author. The data are not publicly available due to privacy restrictions.

## Abbreviations

The following abbreviations are used in this manuscript:

OM	Otitis media multidisciplinary
OME	Otitis media with effusion
TT	Tympanostomy tube
TUT	Tympanostomy U-Tube
